# Determination of IL-23 receptor expression and gene polymorphism (rs1884444) in Iranian patients with ankylosing spondylitis

**DOI:** 10.1186/s41927-024-00383-w

**Published:** 2024-04-11

**Authors:** Atiyeh Mellati, Samaneh Soltani, Tohid Kazemi, Nooshin Ahmadzadeh, Maryam Akhtari, Elham Madreseh, Ahmadreza Jamshidi, Elham Farhadi, Mahdi Mahmoudi

**Affiliations:** 1 Immunology Research Center, Tabriz University of Medical Sciences, Tabriz, Iran; 2 Department of Immunology, Faculty of Medicine, Tabriz University of Medical Sciences, Tabriz, PO-Box: 5165683146, Iran; 3 Rheumatology Research Center, Shariati Hospital, Tehran University of Medical Sciences, Tehran, PO-Box: 1476943313, Iran; 4 Tobacco Prevention and Control Research Center, National Research Institute of Tuberculosis and Lung Diseases (NRITLD), Shahid Beheshti University of Medical Sciences, Tehran, Iran; 5 Research Center For Chronic Inflammatory Diseases, Tehran University of Medical Sciences, Tehran, Iran

**Keywords:** Ankylosing spondylitis, Gene expression, IL-23 receptor, ARMS-PCR

## Abstract

**Background:**

Through investigating genetic variations, it has been demonstrated that single nucleotide polymorphisms (SNPs) in the IL-23 receptor (*IL23R*) gene have a critical role in the pathophysiology of ankylosing spondylitis (AS). Here, we investigated whether the *IL23R* variant (rs1884444) is associated with AS in the Iranian population.

**Methods and material:**

In this research, we analyzed rs1884444 in a group of 425 patients with AS and 400 matched controls. For DNA extraction, the phenol/chloroform technique was utilized. Peripheral blood mononuclear cells (PBMCs) were obtained from the whole blood of 39 patients and 43 healthy controls and total RNA was extracted. Genotyping was performed by amplification-refractory mutation system (ARMS)–PCR method. Afterward, the expression level of *IL23R* was analyzed by the real-time quantitative (Q)-PCR method.

**Results:**

We observed no significant association between the distribution of alleles and genotypes of rs1884444 and susceptibility to AS. In addition, the expression level of *IL23R* did not differ between PBMCs from AS patients compared to the control group (*P* = 0.167). Furthermore, the relative expression level of *IL23R* was positively correlated with the BASDAI (*P* < 0.01) and BASFI (*P* < 0.05) scores of the patients.

**Conclusion:**

It appears that *IL23R* polymorphism (rs1884444) and the level of gene expression might not contribute to the susceptibility to AS in the Iranian population. The correlation of *IL23R* expression with the level of BASDAI and BASFI scores in patients may be due to the role of the IL-23/IL-23R signaling cascade in inflammation and exert a critical role in the development of AS.

## Introduction

Spondyloarthropathy (SpA /also called spondyloarthritis) is a group of inflammatory rheumatic diseases of which the most clinical highlight is inflammation of the spine. Ankylosing spondylitis (AS), a subcategory and the most notable part of seronegative SpA is a polygenic chronic progressive inflammatory arthritis that can progress to significant functional disability. It predominantly affects the sacroiliac and spinal joints and can result in ankylosis and fibrosis of the affected tissues [1]. Males are more affected than females: with a ratio of approximately 2–3:1 and most patients develop the primary signs before the age of 30 [2]. However, the precise pathogenesis of AS is yet unknown, genome-wide association studies (GWAS) have implicated a variety of risk factors for the disease, including major histocompatibility complex (MHC) genes, several innate immune-related pathways, and several non-MHC genes [3]. Accumulating studies have provided evidence showing elevated levels of some pro-inflammatory cytokines, including interleukin (IL)-1β, interferon (IFN)-γ, tumor necrosis factor (TNF)-α, IL-6, IL-17, and IL-23 may be involved in the AS pathogenic process [4].

Interleukin-23 (IL-23), a heterodimeric proinflammatory cytokine, is a potential non-MHC candidate factor that is strongly involved in AS pathogenesis. IL-23 is a member of the IL-12 cytokine family and structurally is made of the IL-23-specific p19 (IL-23p19) and a p40 (IL-12p40) subunits (the latter is shared with IL-12). The IL-23 receptor (IL-23R) is a member of the type I cytokine receptors and its gene spans 92 kb on chromosome 1p31 and comprises 11 exons [5, 6]. The encoded peptide pairs with the β1 subunit of IL-12 (IL-12Rβ1) and forms a specific heterodimeric complex of the IL-23R [7].

IL-23 is indicated as a master mediator that regulates both the innate and adaptive immune responses [8]. Several polymorphisms in the *IL23R* gene are strongly associated with the incidence and susceptibility to organ-characteristic autoimmune diseases, such as inflammatory bowel disease (IBD) [9], rheumatoid arthritis (RA) [10], psoriasis [11], and multiple sclerosis (MS) [12], but not with systemic autoimmune diseases such as systemic sclerosis (SSc) [7, 13], and systemic lupus erythematosus (SLE) [14, 15]. These implied that IL-23R may mostly participate in regulating local inflammation but not systemic inflammation [6].

Previous studies demonstrated that IL-23R is a potential risk factor for AS. Various studies have provided evidence showing that several polymorphisms in the *IL23R* gene (e.g., rs11209032, rs11209026, rs7517847, rs2201841) have correlated with the risk of AS [16, 17]. According to the NCBI SNP database, rs1884444 G/T is considered a non-synonymous G/T single nucleotide polymorphism (SNP) within exon 2 of the *IL23R* gene. Rs1884444 G/T results in the amino acid substitution of glutamine (Gln) by histidine (His) (Gln3His; rs1884444) at the extracellular domain of the receptor and therefore influences the activity and affinity to IL-23 [18].

This study has planned to detect the possible association of rs1884444 *IL23R* gene polymorphism with AS susceptibility in an Iranian population, as well as its effect on disease severity. Furthermore, we evaluated the messenger RNA (mRNA) expression level of IL-23R in peripheral blood mononuclear cells (PBMCs) from AS patients and compared it with normal individuals.

## Materials and methods

### Study population

In this case-control research, the subjects were comprised of 425 AS patients, whose definitive diagnosis had been confirmed based upon the modified New York criteria [19]. The disease activity parameters of the AS patients were evaluated using the Bath ankylosing spondylitis disease activity ındex (BASDAI) [20], the Bath ankylosing spondylitis functional index (BASFI) [21], and the Bath ankylosing spondylitis metrology index (BASMI) [22]. For control group 400 matched healthy controls who had no known history of autoimmune or immunological disorders were enrolled for this study. Before the study, all the participants were asked to sign the written informed consent. The ethics committee of the Tehran University of Medical Sciences approved the current research (IR.TUMS.VCR.REC.1398.886).

### PBMCs preparation and RNA and DNA extraction

Ten millimeters of venous blood were taken from each participant through EDTA-anticoagulated venoject tubes. DNA was extracted from whole blood using the standard proteinase K digestion- phenol/chloroform extraction method [23].

Besides, 39 AS patients and 43 matched healthy participants were randomly selected from participants, and PBMCs were isolated from human whole blood using the Ficoll-Hypaque density gradient centrifugation approach (innotrain, Germany). Total cellular RNA was extracted from freshly isolated PBMCs via a High Pure RNA Isolation Kit (Roche, Germany) following the manufacturer’s recommendations. The concentration and purity of genomic DNA and total RNA samples were quantified by NanoDrop 2000 C spectrophotometer (Thermo Fisher Scientific, USA).

### Primer design and *IL23R* rs1884444 polymorphism

The amplification-refractory mutation system polymerase chain reaction (ARMS-PCR) [24] method was selected to determine the presence of the T > G point mutation in the *IL23R* gene. To design the assay, three primers including two reverse (mutant and wild-type) and one forward common primer (Table 1) were designed by a web-based allele-specific PCR (WASP) assay designing tool. ARMS-PCR was conducted in a final volume of 15 μL containing 1.5 μL of genomic DNA, 7.1 μL distilled water, 0.25 μL 10 mM dNTP (Roje Technologies Co., Iran), 1.2 μL of each specific primer (10 pmol/lL), 2.5 μL10X PCR buffer (Roje Technologies Co., Iran), 0.65 μL 1.5 mM MgCl_2_ (Roje Technologies Co., Iran), and 0.3 μL (5 U/ μL) Taq DNA polymerase buffer (Roje Technologies Co., Iran). Two internal control primers with 0.1 μL volume (10 pmol/lL) in each reaction were also utilized. Amplified products were analyzed by electrophoresis in 2% agarose gel stained with DNA-safe staine and were observed through a UV transilluminator. PCR product sequencing (Direct Sanger sequencing) was performed by 3730xl DNA Analyzer (Applied Biosystems) using big dye terminator.

**Table 1:** Primers used in ARMS and realtime PCR for *IL23R* gene rs1884444 genotyping and relative expression

	Primer Name	Primer Sequence	Tm (°C)	Amplicon size (bp)
ARMS-PCR	Common Forward	5’- TCCTCCCTAATCAAAGGTTCCCATC-3’	61.88	181
	WT Reverse Primer	5’- CTATTACTGCATCCCATTGAATAGTGATA-3’	59.27	
	Mutant Reverse Primer	5’- CTATTACTGCATCCCATTGAATAGTGATC-3’	60.18	
	GH (5F)	5’- TGCCTTCCCAACCATTCCCTTA-3’	61.65	434
	GH (5R)	5’- CCACTCACGGATTTCTGTTGTGTTTC-3’	62.85	
	Sequencing Primer	5’- GGGCTGTCTAGAAGGGAAATTTGAG-3’	61.38	431
Expression	*IL-23R* (F)	5’-ACATGCTTCTATGTACTGCACTG-3’	58.81	184
	*IL-23R* (R)	5’-TGTGTCTATGTAGGTGAGCTTCC-3’	59.55	
	*β2M* (F)	5’-CCTGAATTGCTATGTGTCTGGG-3’	59.05	244
	*β2M *(R)	5’-TGATGCTGCTTACATGTCTCGA-3’	59.83	

Abbreviations: ARMS-PCR: Amplification Refractory Mutation System PCR; Tm: melting temperatures; WT: Wild type; GH: Growth hormone; IL-23R: Interleukin 23 receptor; *β2M*: Beta-2 microglobulin

### Reverse transcription and complementary DNA synthesis

First-strand complementary DNA (cDNA) was polymerized from the RNA of cells using the RT-ROSET kit (Roje Technologies Co., Iran), under the manufacturer’s manuals. In summary, RT-PCR was performed in a 20 μL volume per tube containing isolated RNA (150 ng/μL), 10 μL RTMix (2X), 2 μL of Mixzym, and remaining RT-PCR Grade Water.

### Real-time quantitative polymerase chain reaction

Primers for *IL23R* and β2-microglobulin (*β2M*), which we selected as a reference gene, were designed on the website (https://pga.mgh.harvard.edu/). Gene expression analysis was carried out with specific primers for *IL23R* and *β2M*. Quantitative RT-PCR was performed on a StepOne real-time PCR detection system using the SYBR Green detection mix (Applied Biosystems). Each reaction mixture contained master mix, cDNA, forward and reverse primers, and nuclease-free water. Gene expression values were evaluated by the comparative CT method as described by Schmittgen and Livak [25]. The relative expression of the target gene was obtained using the following equation: relative mRNA expression = (2^−ΔCt^) × 10^3^. The mRNA expression level was normalized to *β2*M.

### Statistical analysis

All statistical analysis was done using SPSS version 22 (SPSS, Chicago, IL, USA) and GraphPad Prism version 6 (La Jolla, CA, USA) software. Group comparisons of continuous variables were analyzed out through the independent sample t-test. Mann-Whitney U non-parametric tests were performed to compare the gene expression data between groups. The correlations between *IL23R* mRNA expression level and SNP expression profiles or clinical indices were determined using Spearman’s correlation. All results were shown as mean ± standard deviation (SD) and a *p*-value of less than 0.05 was considered statistically significant.

## Results

Table 2 depicts the clinical specifications of study subjects and their values as mean ± standard deviation (SD). For genotyping, the male/female ratio in AS group (425 patients; mean age: 38.89 ± 10.095 years) and healthy controls (400 individuals; mean age: 38.37 ± 9.796 years) were 3.3 and 3.1, respectively. In addition, BASMI (4.18 ± 1.89), BASDAI (4.76 ± 2.49), and BASFI (3.87 ± 2.574) were reported for AS patients. Due to the limitation in the expression part, only the data of 39 patients (66.7% male, 33.3% female) and 43 controls (74.4% male, 25.6% female) were analyzed. Also, in the Expression study, BASMI (4.95 ± 1.83), BASDAI (4.63 ± 2.07), and BASFI (3.58 ± 2.52) were reported for AS subjects.

**Table 2:** Statistical comparisons of characteristics between AS patients and healthy controls

	Characteristics		AS Patients	Healthy Controls	*P*-value
Genotyping	Age (Year)^*^		38.89 ± 10.095	38.37 ± 9.796	0.461
	Sex^+^	Male	76.70%	75.50%	0.744
		Female	23.30%	24.50%	
	Clinical Data	BASMI	4.18 ± 1.89	-	NA
		BASDAI	4.76 ± 2.49	-	NA
		BASFI	3.87 ± 2.574	-	NA
Expression	Age (Year)^*^		41.10 ± 10.91	38.84 ± 10.35	0.338
	Sex^+^	Male	66.70%	74.40%	0.475
		Female	33.30%	25.60%	
	Clinical Data	BASMI	4.95 ± 1.83	-	NA
		BASDAI	4.63 ± 2.07	-	NA
		BASFI	3.58 ± 2.52	-	NA

*Indicated as Mean ± SD; + Indicated as n/N; NA; BASMI: Bath Ankylosing Spondylitis Metrology Index; BASDAI: Bath Ankylosing Spondylitis Disease Activity Index; BASFI: Bath Ankylosing Spondylitis Functional Index; Not applicable

### Alleles and genotypes frequencies of *IL23R* rs1884444 variant

The distribution of genotypes (GT/TT vs. GG) and allele frequencies of rs1884444 (G/T) of *IL23R* in AS and controls are presented in Table 3. The frequency of genotypes in the control group did not diverge from Hardy–the Weinberg equilibrium (HWE) (*p* = 0.091). We failed to find any remarkable differences in allele frequencies or genotype variations between cases and controls. Although the odds ratio of the disease in the individuals who have T allele is lower than those who have G allele (95% CI; 0.96 (0.78–1.17)), this rate is not statistically significant (*p* = 0.66).

**Table 3:** Alleles and genotypes distribution of rs1884444 variant in AS and healthy groups

SNP	Allele /Genotype	AS Patients (*N* = 425) N (%)	Controls (*N* = 400) N (%)	OR (95% CI)	*P* value
rs1884444	G	559 (65.76)	518 (64.75)	**Reference**	
	T	291 (34.24)	282 (35.25)	0.96 (0.78–1.17)	0.66
	GG	161 (37.88)	160 (40.00)	**Reference**	
	TT	27 (6.36)	42 (10.50)	0.64 (0.37–1.09)	0.097
	TG	237 (55.76)	198 (49.50)	1.19 (0.89–1.59)	0.24
	TT + TG	264 (62.12)	240 (60.00)	1.09 (0.83–1.45)	0.53
HWE	0.091				

Abbreviations: AS: Ankylosing Spondylitis; OR: Odd Ratio; CI: Confidence Interval; HWE: Hardy-Weinberg equilibrium; ^*^P-value < 0.05

**Fig. 1:**
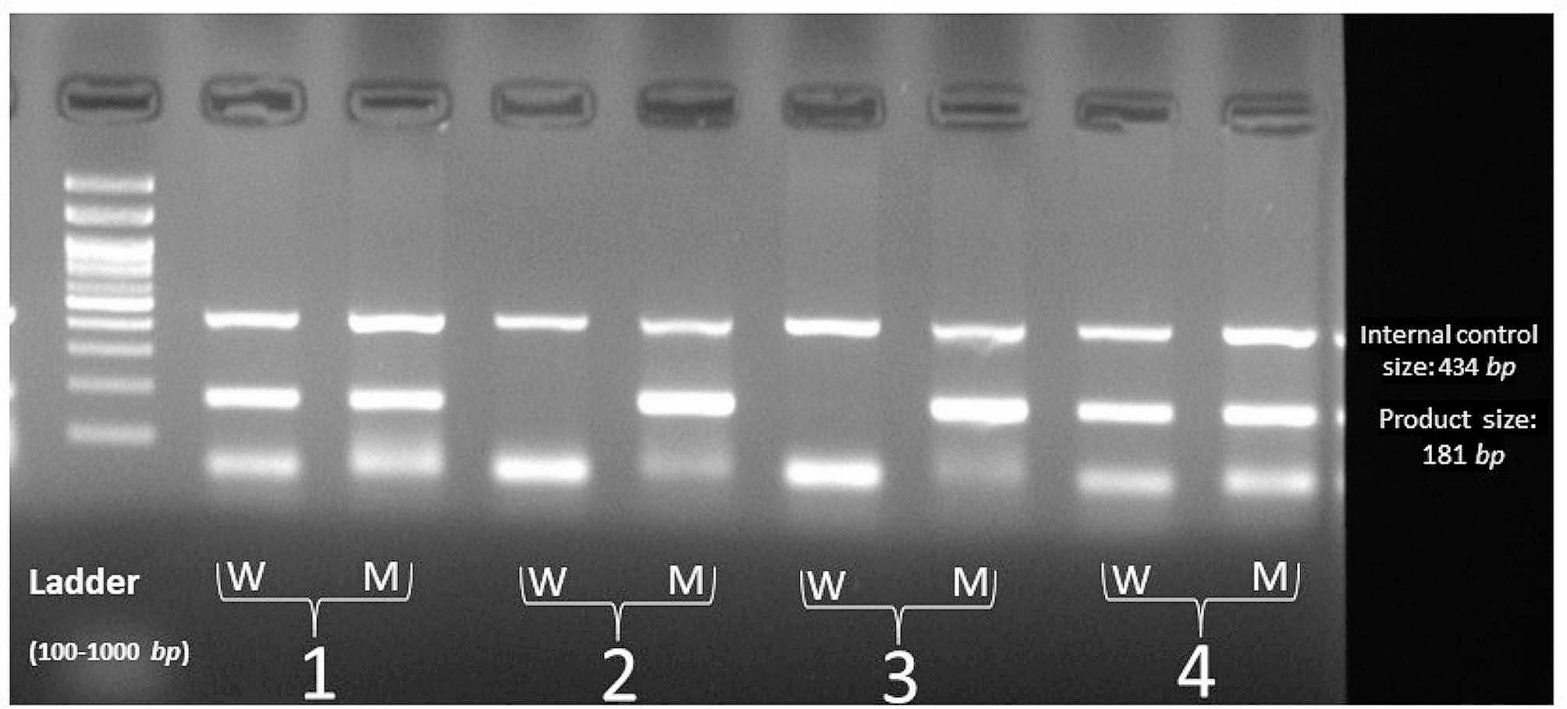
PCR products from 4 various samples. Numbers 1 and 4 are heterozygous (GT) for IL23R/rs1884444; Numbers 2 and 3 are homozygous (GG) for mutant alleles for IL23R/rs1884444. Abbreviations: M; mutant, W; wild

Compared to individuals who have the GG genotype, the odds ratio of the disease is lower in those with the TT genotype (OR (95% CI; 0.64 (0.37–1.09), while the individuals with the TG genotype (OR (95% CI; 1.19 (0.89–1.59)) and those with the TT + TG genotype (OR (95% CI; 1.09 (0.83–1.45)) have a higher chance of developing the disease. Figure 1 demonstrates the PCR products from 4 various samples.

### Evaluation of the mRNA expression level of *IL23R*

Our study results showed the downregulation of mRNA expression level of *IL23R* in AS patients (0.19 ± 0.20) in comparison to the control group (0.26 ± 0.23) however, it was not statistically significant (Fold change = 0.72; *p* = 0.167) (Table 4).

**Table 4:** Comparison of *IL-23R* expression level between AS patients and healthy controls

Expression Level	Controls (*N* = 43)	AS patients (*N* = 39)	FC	P- value	95% CI	
					Lower	Upper
*IL23R*	0.26 ± 0.23	0.19 ± 0.20	0.72	0.167	-0.165	0.291

Abbreviations: FC: fold change; all of the expression values expressed as Mean ± SD, CI: Confidence interval

### The correlation of *IL23R* expression level with clinical parameters

We assessed the relationship between *IL23R* mRNA expression level in PBMCs and the the activity indexes of AS disease, including BASDAI, BASFI, and BASMI. When correlation analysis was applied to the AS subjects, it was seen that the mRNA expression level of *IL23R* was positively correlated with the BASDAI (*r* = 0.424^**^; *p* < 0.01) and BASFI (*r* = 0.338; *p* < 0.05) scores of patients however, it did not show any significant correlation with the BASMI score (*r*=-0.510) (Table 5).

**Table 5:** Matrix of Spearman’s correlation coefficient between the level of AS clinical characteristics and *IL-23R* mRNA expressions in the patients

	IL-23R	BASMI	BASDAI	BASFI
IL23R	1	-0.51	0.424^**^	0.338^*^
BASMI	-	1	0.253^***^	0.415^***^
BASDAI	-	-	1	0.698^***^
BASFI	-	-	-	1

Abbreviations: IL-23R: Interleukin 23 receptor; BASMI: Bath Ankylosing Spondylitis Metrology Index; BASDAI: Bath Ankylosing Spondylitis Disease Activity Index; BASFI: Bath Ankylosing Spondylitis Functional Index; * *p* < 0.05; **p < 0.01; ***p < 0.001

### The correlation of rs188444(G/T) variant and expression level *IL23R*

**Fig. 2:**
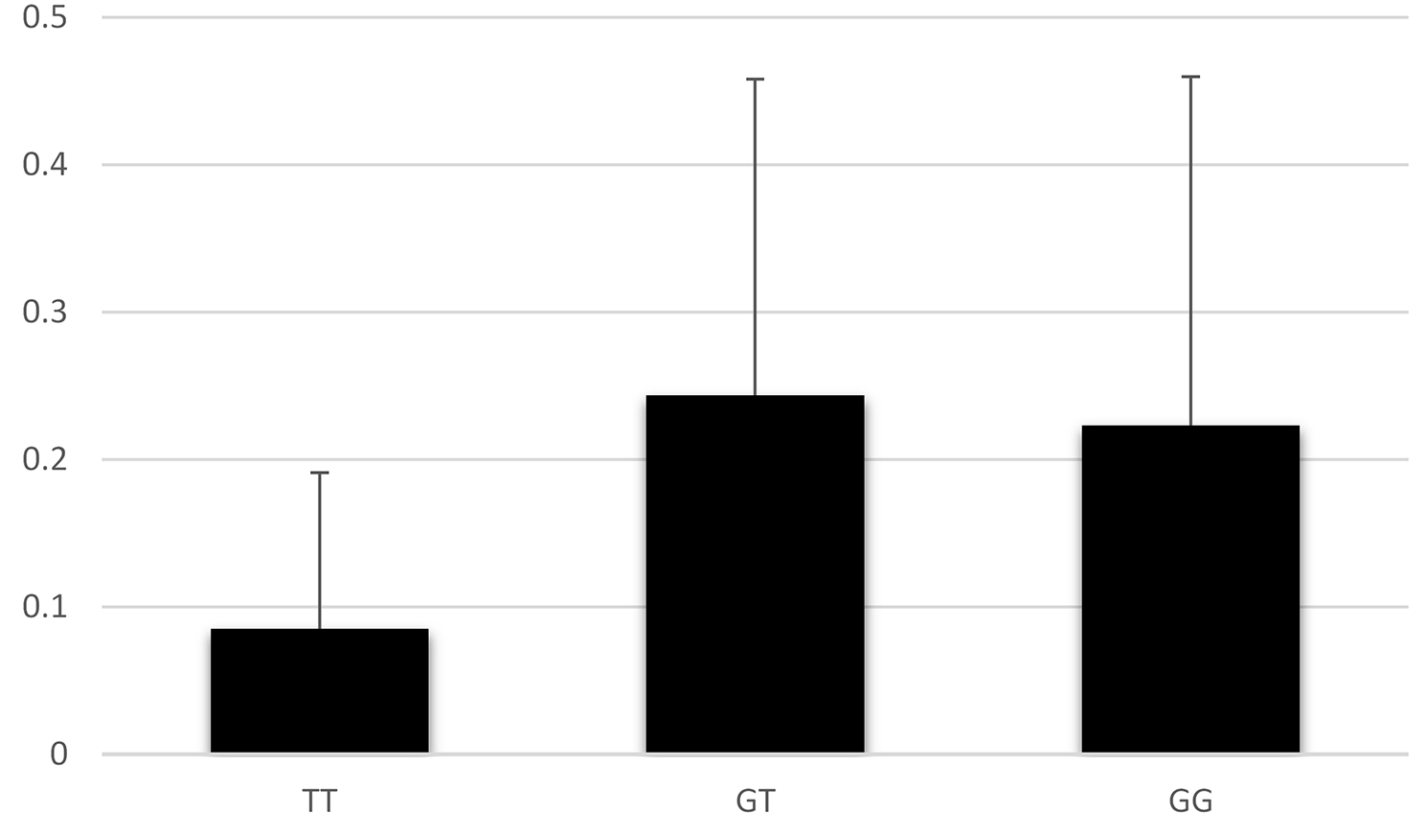
Comparison of IL-23R expression level (rs1884444) among TT, TG, and GG genotypes

*IL23R* gene expression level was evaluated in different rs1884444 genotypes in patients and controls. Analysis of this data showed that different genotypes (TT/GG/GT) of this SNP did not affect the *IL23R* expression level and no significant relationship was found between them (*p* = 0.26). In particular, those possessing the TT genotype were observed to have the lowest levels of IL-23R (Fig. 2).

## Discussion

Ankylosing spondylitis is a progressive multi-system inflammatory arthritis with an estimated prevalence of 0.1–0.9% in Caucasian populations [26]. The exact pathogenesis still remains unexplained and subject to extensive research. A number of studies have shown that certain cytokines have a pivotal role in the activation of the immune system in AS pathogenesis [8]. IL-23, which is crucial for the survival and expansion of Th17 lymphocytes can promote IL-17 production in memory T cells and acts through IL-23R [27]. The Th17 pathway which is mediated by IL-23/IL-23R could induce inflammation and have a crucial role in the pathogenesis of AS [28]. IL-23 affects IFN-*γ* secretion by T lymphocyte and natural killer (NK) cells. It can activate memory T lymphocytes, induce type I immune responses, and by inducing the production of proinflammatory cytokines, upregulate inflammation [14].

There is a growing body of research that addressed the association of certain polymorphisms in *IL23R* with AS susceptibility in different populations, but there was no consensus reached [29]. These discrepancies in the results of various studies might be attributed to the different ethnicities, genetic backgrounds, and different sample sizes. In this study, we first assessed the possible association of rs1884444 of *IL23R* with AS susceptibility in an Iranian population. The results of genotype analysis from this study did not show any significant relationship between the frequency of alleles and the distribution of genotypes and the risk of AS. In a research by Yang et al., 486 AS patients and 480 healthy controls were investigated for *IL-23R* gene polymorphisms. Patients with genotype CC and allele C on SNP rs6693831 (C > T) represented a decreased risk of AS. Besides, individuals with the genotype TT and allele T on SNP rs1884444 (T > G) revealed a significantly lower risk of AS. The results indicated a significant association between rs6693831 and rs1884444 polymorphisms and AS susceptibility. Additionally, patients with rs6693831 CT genotype represent significantly higher levels of CRP in comparison to those with CC and TT genotypes [28].

In a study by Daryabor et al., consisting of 294 AS patients and 352 healthy controls in an Iranian population, five variants including, rs1004819, rs11209026, rs1495965, rs11209032, and rs11465804 in *IL23R* were investigated. The results demonstrated that rs1004819 has a strong association with AS and that the remaining four SNP alleles are not related to AS susceptibility. Furthermore, they found no correlation between studied SNPs and BASMI, BASFI, and BASDAI scores of patients [30].

In a study in the Korean population by Sung et al., 451 AS patients and 392 ethnicities matched healthy control were genotyped for *IL23R* polymorphisms. Ten SNP including, rs7517847, rs1004819, rs2201841, rs10489629, rs1343151, rs11465804, rs11209026, rs10889677, rs1495965, and rs11209032 within the *IL23R* gene cluster were genotyped. No *IL23R* SNP was revealed to be associated with AS in the Korean cohort [31].

In a case-control study by Davidson et al., 492 Han Chinese patients with AS in comparison to 846 healthy controls were investigated for 21 SNPs covering *IL23R* (not including rs1884444). No association was found between genotyped SNPs in *IL23R* and AS development [32].

The study by Pimentel-Santos et al., comprised 358 unrelated AS cases and 285 Portuguese-matched healthy controls. Eight SNPs markers including 67,442,801, 67,460,937, 67,475,114, 67,491,717, 67,497,708, 67,478,546, 67,526,096, and 67,512,680 for *IL23R* were analyzed. Two SNPs (rs1004819, and rs10889677) indicated a significant association with AS incidence. These results exhibited that some SNPs in the *IL23R* gene are correlated with susceptibility to AS in the Portuguese population [26].

In 2010, Duan et al. conducted a meta-analysis investigating the possible association between *IL23R* gene polymorphisms and AS susceptibility. Eleven articles, including 13 populations were analyzed. The frequency of rs1004819 and rs11209032 allele A were higher within AS cases than within the controls. The frequency of rs1343151 allele T, rs10489629 allele G, and rs11209026 allele A was lower in the AS group than in the controls. Overall, the results of this meta-analysis suggested that the AS susceptibility is associated with the *IL23R* gene polymorphisms. The protective role may be attributed to rs1343151, rs10489629, and rs11209026 SNPs, while rs1004819 and rs11209032 act as susceptible SNPs [2].

In another meta-analysis performed by Zhong et al., 25 case-control studies (8431 patients and 8972 healthy controls) were included and analyzed for 10 widely studied polymorphisms of *IL23R*. The results showed that carriers of rs1004819, rs2201841, and rs1495965 minor alleles were susceptible to AS, however; the minor alleles of rs134315, rs10489629, rs11465804, and rs11209026 have protective roles against AS [29].

To study the association of SNPs in IL-23R, 138 Chinese Han populations with AS and 129 matched controls were investigated. The results did not reveal any remarkable difference in the genotype and allele frequency of rs1343151 between cases and controls. However, they noted a substantial difference in genotype frequency of rs11209032 and rs6677188 between AS patients and controls. They also investigated the expression of *IL23R* in PBMCs from patients and controls. An elevated level of *IL23R* mRNA was shown in PBMCs of AS patients [33].

We also analyzed the expression of *IL23R* in PBMCs from AS patients and healthy individuals and investigated the correlation of this expression with the score of clinical manifestations of patients. We did not observe a statistically remarkable difference in the mRNA level of *IL23R* between AS patients and healthy participants. However, the relative expression level of *IL23R* was positively associated with the score of BASDAI and BASFI indexes of patients. IL-23R is involved in initiating, perpetuating, and accelerating the IL-23/IL-17 inflammatory axis [34]. Thus, this correlation may be due to the role of the IL-23/IL-23R signaling cascade in the Th17 pathway which could induce inflammation and exert a critical role in the AS disease development [28]. Therefore, it is suggested that *IL23R* mRNA level may be related to AS activity and disease symptoms in the Iranian population.

Previous studies reported an elevated level of IL-23 in the serum of AS patients. Chen et al. conducted a study on Chinses population and observed remarkably higher serum levels of IL-17 and IL-23 in patients than in healthy individuals. In the AS cohort study, the score of the BASDAI index had a better correlation with the serum levels of IL-17 or IL-23 than with the level of ESR and CRP [35].

In a small Chinese study conducted by Wang et al., 57 patients with AS and 38 healthy individuals were enrolled. The results demonstrated an increased expression of IL-23 (p19 subunit) mRNA in PBMCs of AS patients in comparison to normal subjects after stimulation with Staphylococcus aureus Cowan I (SAC) [36].

Various candidate polymorphisms in the *IL23R* gene can enhance its expression on different immune cells, and augment the inflammatory condition [30]. The *IL23R* (rs1884444 (T/G)) variant is a missense variant at exon 2 of *IL23R* inducing a substitution of histidine (H) with glutamine (Q) at codon 3 [37]. However, our data showed that the different genotypes and alleles of this polymorphism (rs1884444) did no effect on its expression statistically. Based on a literature review, there were no data available comparing the association of different genotypes of rs1884444 G/T variant and mRNA level of *IL23R* in AS individuals in other populations. Thus, our study is the first, which investigates the relationship between the expression levels of *IL23R* among AS subjects carrying different rs1884444 genotypes.

Considering that in our study, some associations between the variables were not significant, we calculated the power of the study. Since, using G*Power software and estimated proportions of Alleles (T) and genotypes (TT, TG and TT + TG) of rs1884444 between in AS (*n* = 425) and healthy (*n* = 400) groups, so the power analysis was done. Therefore, the estimated powers were 0.51, 0.80, 0.75 and 0.54, respectively. Also, the Power to Compare *IL-23R* expression level between AS patients (*n* = 39) and healthy controls (*n* = 43) was 0.68.

In general, there are susceptibility factors that lead to allelic frequencies of a gene showing an association or lack of association with a disease, including the ethnicity of the population; different sample sizes of both women and men; evaluation of a number of SNPs; type of device and material used; study length; co-existence of other autoimmune diseases; heterogeneity between sets of participants, including genetic and clinical heterogeneity; and different disease activity.

The strengths of the current study are its concise design and the matching of cases and controls. Other strengths of the present study lie in the large sample of clinically well-characterized AS patients with a broad variety of disease activity and severity, and the fact that the controls were selected randomly from the general population.

There are some limitations and caveats concerning the current study, including the budget, ethical considerations, and staffing of specialists also exist that need to be taken into account. Also, in our study, we did not analyze the treatment administered to patients as a possible factor that influences physical outcome, since we did not have reliable data about which patients were on a specific therapy before their inclusion in the study. The lack of information about the treatment could have introduced some bias.

## Conclusion

The *IL23R* gene, a receptor of key immunoregulatory cytokine with pro-inflammatory properties, became one of the most non-HLA candidate genes in several organ-specific autoimmune diseases. The current study suggested that the rs1884444 G/T variant in the *IL23R* gene has no association with AS susceptibility in the Iranian population and also the level of *IL23R* did not differ between AS patients and healthy participants. However, we found a positive correlation between its expression in PBMCs and the score of clinical manifestations of AS patients. Therefore, it seems that further functional studies in a larger number of AS patients with various detection methods in different populations with bigger sample sizes and different SNPs might help to better comprehend the contribution of the *IL23R* gene variants and expression in the occurrence of AS and the incidence of its clinical manifestations in various ethnic populations.

## Data Availability

Data supporting the findings in the present work are available upon reasonable request.

## Abbreviations

***β2M***: β2-microglobulin
ARMS: Amplification-refractory mutation system
AS: Ankylosing spondylitis
BASFI: Bath Ankylosing Spondylitis Functional Index
BASMI: Bath Ankylosing Spondylitis Metrologic Index
CI: Confidence interval
CRP: C-reactive protein
ESR: Erythrocyte sedimentation rate
GWAS: Genome-wide association studies
HLA: Human leukocyte antigen
HWE: Hardy–the Weinberg equilibrium
IBD: Inflammatory Bowel Disease
IFN: Interferon
IL-23R: IL-23 receptor
IL: Interleukin
MHC: Major histocompatibility complex
mRNA: Messenger RNA
MS: Multiple Sclerosis
PBMCs: Peripheral blood mononuclear cells
RA: Rheumatoid arthritis
SLE: Systemic Lupus Erythematosus
SNP: Single nucleotide polymorphism
SpA: Spondyloarthropathy
TNF: Tumor Necrosis Factor
WASP: Web-based allele-specific PCR
